# Quantification of Treatment Effect Modification on Both an Additive and Multiplicative Scale

**DOI:** 10.1371/journal.pone.0153010

**Published:** 2016-04-05

**Authors:** Nicolas Girerd, Muriel Rabilloud, Philippe Pibarot, Patrick Mathieu, Pascal Roy

**Affiliations:** 1 INSERM, Centre d’Investigations Cliniques 1433, Université de Lorraine, CHU de Nancy, Institut Lorrain du cœur et des vaisseaux, Nancy, France; 2 Hospices Civils de Lyon, Service de Biostatistiques, Lyon, F-69003, France, Université de Lyon, Lyon, F-69000, France, Université Lyon I, Villeurbanne, F-69100, France, CNRS, UMR5558, Laboratoire de Biométrie et Biologie Evolutive, Equipe Biostatistiques Santé, Villeurbanne, F-69100, France; 3 Department of Medicine, Laval University, Québec, Canada; 4 Department of Surgery, Laval University, Quebec, Canada; Taipei Medical University, TAIWAN

## Abstract

**Background:**

In both observational and randomized studies, associations with overall survival are by and large assessed on a multiplicative scale using the Cox model. However, clinicians and clinical researchers have an ardent interest in assessing absolute benefit associated with treatments. In older patients, some studies have reported lower relative treatment effect, which might translate into similar or even greater absolute treatment effect given their high baseline hazard for clinical events.

**Methods:**

The effect of treatment and the effect modification of treatment were respectively assessed using a multiplicative and an additive hazard model in an analysis adjusted for propensity score in the context of coronary surgery.

**Results:**

The multiplicative model yielded a lower relative hazard reduction with bilateral internal thoracic artery grafting in older patients (Hazard ratio for interaction/year = 1.03, 95%CI: 1.00 to 1.06, p = 0.05) whereas the additive model reported a similar absolute hazard reduction with increasing age (Delta for interaction/year = 0.10, 95%CI: -0.27 to 0.46, p = 0.61). The number needed to treat derived from the propensity score-adjusted multiplicative model was remarkably similar at the end of the follow-up in patients aged < = 60 and in patients >70.

**Conclusions:**

The present example demonstrates that a lower treatment effect in older patients on a relative scale can conversely translate into a similar treatment effect on an additive scale due to large baseline hazard differences. Importantly, absolute risk reduction, either crude or adjusted, can be calculated from multiplicative survival models. We advocate for a wider use of the absolute scale, especially using additive hazard models, to assess treatment effect and treatment effect modification.

## Introduction

Assessment of treatment effect and treatment effect modification is the core of therapeutic clinical research. In both observational and randomized studies, associations with overall survival are mostly assessed on a multiplicative scale using the Cox model. However, clinicians and clinical researchers have an ardent interest in assessing absolute benefit associated with treatments. In addition, we previously reported that the interpretation of the same data can considerably differ when analyses are performed on an additive or on a multiplicative scale [1–5]. Unfortunately, assessing absolute treatment effect is often difficult from the data provided in much of the scientific literature [6].

In previous studies, we investigated the impact of age on the effect of several surgical modalities of coronary revascularization on patient outcome[7–9]. Using Cox models with all-cause mortality as outcome variable, we demonstrated that older patients were less likely to benefit from intensive coronary revascularization such as complete revascularization or bilateral internal-thoracic artery (ITA) graft. This may be explained by the fact that a specific cardiovascular intervention (i.e. intensive coronary revascularization) can only reduce cardiovascular mortality that is targeted by the intervention. In addition, if it is anticipated that aggressive coronary revascularization can decrease acute coronary syndromes, sudden death and acute heart failure, a significant impact of coronary revascularization on death rate from stroke is unlikely. The absence of impact of the intervention on non-cardiovascular mortality and on cardiovascular mortality non-targeted by the intervention translates into a hazard ratio for all-cause mortality closer to one, when the all-cause mortality rate increases, which is the case with increasing age. In contrast, young patients in whom most of the observed mortality is related to the disease treated by the intervention would have a higher all-cause mortality hazard ratio (more distant to 1) than that observed in elderly patients, even if the absolute reduction in hazard is similar.

The absolute difference in risk at a given time can be calculated from either a multiplicative or an additive model. However, the absolute risk difference method estimates the absolute treatment effect at the level of the risk (risk differences) whereas the usual estimation of relative treatment effect is made at the level of the rates (hazard ratios). We believe that assessing both the absolute and relative treatment effect at the level of the rates would result in a more homogenous reporting of results and could be more straightforward and easy to follow for readers.

In additive survival models, the model estimates absolute differences in cumulative hazards. The most widely used additive survival model is the Aalen model. Increasing attention has been paid to this additive hazard model in the field of epidemiology [10,11], most likely largely due to its recent much simpler use in statistical software packages. From both additive and multiplicative models, the effect of explanatory variables can be expressed as a risk difference at a given time or as a number needed to treat (NNT), thus yielding the absolute effect of a given variable on outcome. In the field of therapeutic clinical research, the assessment of absolute risk reduction is of uttermost importance. Indeed, higher absolute risk reduction in some of the subgroups treated with the medication of interest translates into a higher number of prevented deaths for a given number of treated patients in these subgroups. The identification of subsets of patients among whom a large number of deaths can be prevented is typically, as stated above, the goal of clinicians and clinical researchers.

The gold standard for assessing treatment effect is randomized clinical trials (RCT). However, in certain clinical fields such as surgery, RCTs are very difficult to conduct. In these difficult situations, a large body of literature assesses treatment effect from observational studies. In such studies, controlling for treatment attribution bias with propensity score (PS) is one of the preferred methods to assess minimally biased treatment effect. However, both RCTs and observational studies focusing on survival are analyzed with Cox models and the treatment effect is expressed by a hazard ratio. In these studies, the additive hazard modeling or the use of the absolute risk difference could translate into a different quantification of the treatment effect and of treatment effect modification.

To illustrate this putative difference, we have estimated the respective effect of treatment and the effect modification of treatment using a multiplicative hazard model and an additive hazard model in subgroups of patients of different ages in an analysis adjusted for propensity score, in the context of coronary surgery. In addition, we have furthermore estimated the absolute risk difference using a multiplicative hazard model in different age subsets.

## Methods

### Data

A total of 9862 patients who underwent surgery for a first isolated on-pump coronary artery grafting bypass (CABG) at the Quebec Heart and Lung Institute between 1995 and 2008 were included in this analysis. The preoperative and operative data of all patients were prospectively collected and entered in a computerized database. Long-term survival data were obtained from death certificates of the Registry Office of the Quebec Government from which all-cause mortality was analyzed. Information from the Registry Office was available for every patient until December 31st 2009.

### Ethics

All procedures performed in studies involving human participants were in accordance with the ethical standards of the institutional and/or national research committee and with the 1964 Helsinki declaration and its later amendments or comparable ethical standards. For this type of study formal consent is not required. The Quebec Heart and Lung Institute ethical committee approved this study as well as the dispensation of consent for this retrospective analysis. Importantly, within the frame of this study, patient records/information was anonymized and de-identified prior to analysis.

### Statistical methods

Continuous variables are expressed as mean±SD and were compared using t tests for independent samples. Differences in proportion were compared using a Chi^2^ test. The Kaplan-Meier method was used to estimate survivals. Differences between survival curves were analyzed using the log rank test.

### Survival models

In survival models, the hazard is modeled either on an additive or a multiplicative scale. The hazard difference (in an additive model) or the hazard ratio (in a multiplicative model) can be constant or vary with time. In the present study, both the Aalen and Cox models, which are respectively the most commonly used additive and multiplicative survival models[10,12–14], were used as representatives of these models. In both models, the baseline hazard is always a non-parametric time-dependent function.

The multiplicative models can model the hazard ratio associated with a covariate as either a parametric or a non-parametric function of time. After testing for a non-constant effect using the Schoenfeld residuals, if the hazard ratio is deemed to be constant over time, the proportional hazard Cox model can be used.

Likewise, the Aalen model is a nonparametric flexible survival model that can model the hazard difference associated with a covariate as either a parametric or a non-parametric function of time. After testing for a non-constant effect, if the hazard difference is deemed to be constant over time, the additive model with constant hazard difference can be used.

The procedure used to assess the consistency of effect over time in the Cox and Aalen models are reported in **S1 Text**. In this study, given the results provided in **S1 Text**, the Cox proportional hazard model was used to assess the relative effect of covariates on hazard while the constant hazard difference additive model was used to assess the absolute effect of covariates on hazard.

Estimation of cumulative martingale residuals for both the additive and multiplicative models to determine the functional form of continuous variables was efficiently performed with the “Timereg” package of the R software. For dichotomous variables, the visual comparison of survival curves predicted by the constant hazard difference additive model and the proportional hazard Cox model with Kaplan Meier curves (which are fully non-parametric) is the most straightforward approach to assess model fit. The analysis of martingale residuals for the propensity score is presented in **S2 Text** whereas the comparison of the predicted and Kaplan Meier curves are presented in the core of the manuscript.

Additional statistical details and formal information regarding the additive hazard model and the Cox model are provided in **S3 Text**.

### Number needed to treat

The calculation of number needed to treat (NNT) for time-to-event data is usually based on risk (R) differences.[15,16] When investigating treatment effect, NNT(t) to benefit represents the average number of patients who need to be treated to observe one additional event-free patient at the given time **t** compared with the control group.[15] In clinical trials, NNTs(t) are typically calculated with the Kaplan-Meier method.

Next, the following formulas are used:

$$NNT\left(t\right)=\frac{1}{R_{1}\left(t\right)-R_{2}\left(t\right)}=\frac{1}{S_{2}\left(t\right)-S_{1}\left(t\right)}$$

where

*R*_1_(*t*) and *R*_2_(*t*) are the risks at a given time t for group 1 and group 2, and *S*_1_(*t*) and *S*_2_(*t*) are the survival probabilities at a given time t for group 1 and group 2.

NNT(t) can be evaluated from the additive and the multiplicative model. Indeed, from the two considered models, absolute risk reduction can be determined at a given time. From both models, baseline cumulative risk can be estimated. In a second step, we can determine the probability of outcome occurring at a given time point if each subject in the cohort was treated and if each subject was untreated, based on the covariates in the regression model. These patient-level probabilities are then averaged to determine the average probability of the occurrence in the considered population of an event at a given time point if all subjects were treated and if all subjects were untreated. The difference between these two probabilities provides an absolute risk reduction which, in this instance, is a marginal effect, irrespectively of the model used for its calculation. Austin has used this method previously for logistic regression and Cox models.[17] The associated number needed to treat can be derived from this absolute risk reduction.

### Survival analysis strategy

In order to accurately assess treatment effect and treatment effect modification on an additive scale and on a relative scale, a constant hazard difference additive model and a proportional hazard Cox model were both performed.

In order to identify an interaction between the considered treatment (bilateral ITA) and age on an additive scale from a multiplicative model, absolute risk differences were calculated from the propensity score-adjusted proportional hazard Cox model.

### Propensity score (PS)

A propensity score was constructed in order to better control selection bias potentially related to the use of bilateral ITA graft.[18,19] The propensity score representing the likelihood of having a bilateral ITA was calculated for each patient by using a logistic model with bilateral ITA graft as the dependent variable. Variables included in the logistic regression analysis as independent variables were selected a priori, on the basis on their known or suspected association with both bilateral ITA graft and operative/long-term mortality.

Given that the martingale residuals indicated a poor model fit for both the additive and multiplicative models with PS used, as a linear variable, a cubic spline with 4 knots was used to model the functional form of the effect of PS. Boundary knots, outside of which only the linear portion of the spline was considered, were placed at the 5^th^ and 95^th^ percentiles. The model fit (see **S2 Text**) was acceptable after modeling the effect of PS with a spline and no significant interaction with time was identified for the type of coronary surgery, patient age and PS as well as the interaction between age and PS.

A probability value <0.05 was considered significant. All statistical analyses were performed with SPSS 21 (SPSS Inc., Chicago, IL) and R Software (The R Foundation for Statistical Computing).

## Results

### Characteristics of patients with and without complete revascularization

In the study population (n = 9862), 1070 patients (10.8%) had bilateral ITA grafting (Table 1). These latter patients were less likely to have comorbidities (diabetes, hypertension, renal failure, peripheral vascular disease, chronic obstructive pulmonary disease), left ventricular ejection fraction (LVEF) <50% or previous myocardial infarction than patients with unilateral ITA grafting. Age was an important determinant of bilateral ITA graft: 724 (22.5%) patients <60 years old comparatively to only 346 (5.2%) patients ≥60 years old were treated with bilateral ITA graft.

**Table 1:** Demographic, clinical and surgical data according to the use of unilateral versus bilateral ITA graft.

	Whole population n = 9862	Unilateral ITA graft n = 8792	Bilateral ITA graft n = 1070	p-value	p-value adjusted for PS
**Demographic and clinical data**					
Age, years	63.4±9.1	64.3±8.8	55.6±8.1	<0.001	0.06
Female gender	21.0%	22.3%	9.7%	<0.001	0.60
Body mass index >30 kg.m^-2^	27.4%	28.3%	20.3%	<0.001	0.94
Diabetes	31.2%	33.5%	12.2%	<0.001	0.25
Hypertension	63.1%	64.7%	49.1%	<0.001	0.71
Renal failure	5.7%	6.2%	1.5%	<0.001	0.54
Peripheral vascular disease	16.3%	17.1%	9.7%	<0.001	0.92
COPD	11.7%	12.5%	5.7%	<0.001	0.76
Left ventricular ejection fraction <50%	18.2%	18.7%	14.1%	<0.001	0.95
Previous myocardial infarction	50.6%	51.0%	47.7%	0.04	0.94
Previous stenting	8.0%	8.1%	7.5%	0.51	0.98
Three-vessel disease	53.1%	52.9%	53.1%	0.85	0.99
**Surgical data**					
Urgent surgery	26.3%	26.6%	23.6%	0.03	0.94
CPB time, min	75.5±23.5	74.9±23.3	80.7±24.5	<0.001	<0.001
Number of grafts	3.5±1.0	3.5±1.0	3.7±1.0	<0.001	0.82

ITA, internal thoracic artery; COPD, chronic obstructive pulmonary disease; CPB, cardiopulmonary bypass

In multivariate analysis, older age, female gender and presence of a BMI>30, diabetes, hypertension, renal failure and LVEF<50% were independently associated with an increased probability for bilateral ITA graft (Table 2). The logistic model presented in Table 2 was used for the calculation of the propensity score of each patient.

**Table 2:** Predictive model of the use of bilateral ITA graft vs. unilateral ITA graft.

Variables	OR	95% of Confidence Interval	p-value
Age	0.90	0.89 to 0.91	<0.001
Female gender	0.57	0.45 to 0.71	<0.001
Body mass index >30 kg.m^-2^	0.64	0.54 to 0.77	<0.001
Diabetes	0.35	0.28 to 0.42	<0.001
Hypertension	0.84	0.73 to 0.97	0.02
Renal failure	0.37	0.22 to 0.64	<0.001
Peripheral vascular disease	0.88	0.70 to 1.11	0.28
COPD	0.65	0.49 to 0.87	0.003
Left ventricular ejection fraction <50%	0.80	0.65 to 0.99	0.04
Previous myocardial infarction	0.86	0.74 to 0.99	0.04
Previous stenting	0.94	0.72 to 1.23	0.65
Three-vessel disease	1.01	0.87 to 1.18	0.95
Number of grafts	1.40	1.30 to 1.51	<0.001
Urgent surgery	0.85	0.72 to 1.01	0.06

ITA, internal thoracic artery; COPD, chronic obstructive pulmonary disease

### Survival probabilities derived from the Kaplan-Meier method and from the unadjusted multiplicative and additive models

The mean follow up was 6.6±3.6 years. Overall, 1466 patients (14.9%) died during follow-up.

**Fig 1:**
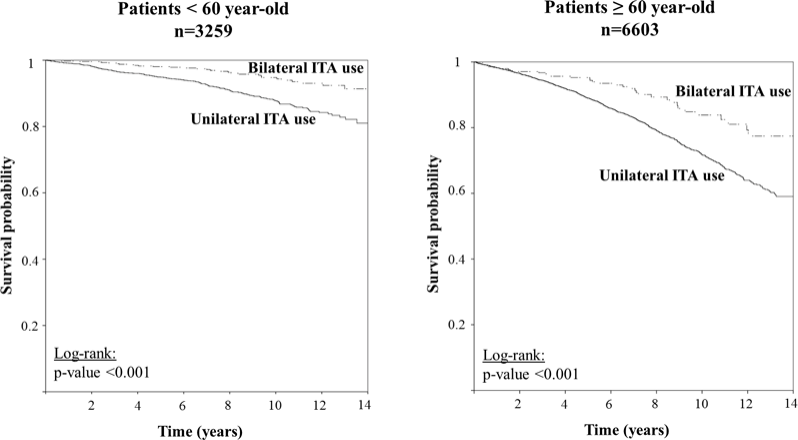
Long-term survival in patients with unilateral or bilateral ITA use in subsets of age (<60 and ≥60 years old). ITA, internal thoracic artery.

Ten-year survival assessed with the Kaplan-Meier method in patients with and without bilateral ITA graft was better in the subset of patients <60 years old than in the subset of patient ≥60 years old (94.8±1.1% vs. 87.8±0.9% in patients <60 and 83.8±2.9% vs. 71.8±0.8% in patients ≥60) (Fig 1).

**Fig 2:**
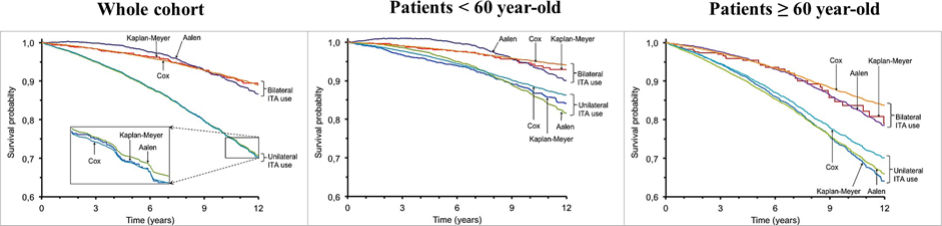
Survival curves generated with the Kaplan-Meier Method, the Aalen model and the Cox model. ITA, internal thoracic artery.

With the Kaplan-Meier method set as reference, the additive model overestimated the survival probability of patients treated with bilateral ITA graft in the whole population during the first part of the follow-up while underestimating the survival probability in the last part of the follow-up (Fig 2, left panel). However, when taking into account age subsets, the survival probabilities calculated with the additive model were found to be closer to those calculated with the Kaplan Meier method in patients aged ≥60 (Fig 2, center and right panels).

### Interaction between bilateral ITA graft and age

Using the univariable Cox model, a lower relative hazard reduction was observed with bilateral ITA in older patients when considering age as a continuous variable with a linear effect on the logarithm of the baseline hazard (HR for interaction Bilateral ITA graft*Age (per 1 year) = 1.04, 95% CI: 1.01 to 1.07, Table 3). Using the univariable additive model, a greater hazard reduction was found with bilateral ITA in the older patients (∆ for interaction = -0.34, 95% CI: -0.68 to 0.00, Table 3).

**Table 3:** Comparison of the estimation of the interaction between age and the use of bilateral ITA graft on the risk for all-cause death in the Cox Proportional Hazards Model and the Aalen Additive Hazards Model.

	Cox Proportional Hazards Model			Aalen Additive Hazards Model		
	Hazard ratio	95% CI	p	Nb of additional all-cause deaths /1000 patients*year	95% CI	p
**Unadjusted models**						
**Model in the whole population with age as a continuous linear variable**						
Age (for a one year increase)	1.06	1.05 to 1.07	<0.001	1.20	1.06 to 1.34	<0.001
Bilateral ITA graft vs. unilateral ITA graft (centered at 60 y.o.)	0.55	0.42 to 0.70	<0.001	-8.25	-11.66 to -4.84	<0.001
Bilateral ITA graft vs. unilateral ITA graft*Age	1.04	1.01 to 1.07	0.01	-0.34	-0.68 to 0.00	0.05
**Analysis according to age categories**						
Bilateral ITA graft vs. unilateral ITA graft when Age < = 60	0.39	0.27 to 0.56	<0.001	-8.74	-11.29 to -6.19	<0.001
Bilateral ITA graft vs. unilateral ITA graft when Age 60 to 70	0.57	0.38 to 0.85	0.006	-10.70	-16.62 to -4.78	<0.001
Bilateral ITA graft vs. unilateral ITA graft when Age >70	0.83	0.43 to 1.61	0.59	-6.05	-28.20 to 16.10	0.59
Models adjusted for propensity score*						
**Model in the whole population with age as a continuous linear variable**						
Age (for a one year increase)	1.01	1.00 to 1.02	0.17	0.17	-0.06 to 0.40	0.15
Bilateral ITA graft vs. unilateral ITA graft (centered at 60 y.o.)	0.70	0.54 to 0.91	0.007	-3.60	-7.07 to -0.13	0.04
Bilateral ITA graft vs. unilateral ITA graft*Age	1.03	1.00 to 1.06	0.05	0.10	-0.27 to 0.46	0.61
**Analysis according to age categories**						
Bilateral ITA graft vs. unilateral ITA graft when Age < = 60	0.59	0.40 to 0.86	0.006	-4.46	-7.13 to -1.79	0.001
Bilateral ITA graft vs. unilateral ITA graft when Age 60 to 70	0.86	0.57 to 1.31	0.49	-2.25	-8.37 to 3.87	0.47
Bilateral ITA graft vs. unilateral ITA graft when Age >70	0.84	0.42 to 1.69	0.63	-6.05	-25.85 to 13.75	0.55

*Propensity score was entered in the models as a linear spline with 4 knots

ITA, internal thoracic artery; y.o., years old

Upon adjusting for PS, the Cox model still yielded a lower relative hazard reduction with bilateral ITA in the older patients (HR for interaction = 1.03, 95% CI: 1.00 to 1.06) whereas the PS-adjusted additive model yielded a similar absolute hazard reduction with increased age (Table 3), which suggests that the effect is additive (i.e. the additive model is the most parsimonious model here) rather than multiplicative.

### Analysis adjusted for PS in three age categories

When adjusting for PS, the association between bilateral ITA graft and survival was weaker in older patients when measured on a relative scale (HR = 0.59, 95% CI: 0.40–0.86 in patients < = 60 vs. HR > 0.80 in patients 60 to 70 and >70). The shape of the association between bilateral ITA graft and survival measured on an additive scale differed, with patients aged >70 having the higher absolute decrease in hazard (delta = -6.05, 95% CI:-25.85 to 13.75, Table 3).

### NNT from the Cox model according to age subsets

NNTs derived from PS-adjusted predicted survival using the Cox model were remarkably similar at the end of the follow-up in patients aged < = 60 and in patients >70 (NNT at 8 and 10 years = 27.7 and 20.0 in patients aged < = 60 vs. 27.7 and 22.0 in patients aged >70, respectively) (Table 4).

**Table 4:** Absolute risk difference estimations and number needed to treat to benefit provided by propensity score-adjusted Cox Models according to age subsets.

	PS-adjusted Cox Models											
	Subset of patients aged < = 60 PS-adjusted HR = 0.59, 0.40 to 0.86, p = 0.006				Subset of patients aged 60 to 70 PS-adjusted HR = 0.86, 0.57 to 1.31, p = 0.49				Subset of patients aged >70 PS-adjusted HR = 0.84, 0.42 to 1.69, p = 0.63			
	Survival at year N		Absolute risk difference at year N	NNT to benefit	Survival at year N		Absolute risk difference at year N	NNT to benefit	Survival at year N			
Years	Without bilateral ITA	With bilateral ITA			Without bilateral ITA	With bilateral ITA			Without bilateral ITA	With bilateral ITA	Absolute risk difference at year N	NNT to benefit
N = 2	98.52%	99.13%	**0.61%**	**164.1**	97.12%	97.50%	**0.39%**	**258.6**	95.28%	96.01%	**0.73%**	**136.3**
N = 4	96.54%	97.95%	**1.41%**	**70.8**	93.33%	94.20%	**0.88%**	**114.2**	89.22%	90.84%	**1.62%**	**61.6**
N = 6	94.07%	96.47%	**2.40%**	**41.7**	88.72%	90.16%	**1.44%**	**69.4**	82.06%	84.65%	**2.59%**	**38.6**
N = 8	90.96%	94.56%	**3.61%**	**27.7**	83.08%	85.16%	**2.09%**	**47.9**	73.64%	77.24%	**3.61%**	**27.7**
N = 10	87.24%	92.24%	**5.00%**	**20.0**	76.60%	79.36%	**2.76%**	**36.2**	64.42%	68.97%	**4.55%**	**22.0**

Legend: absolute risk was calculated according to the following formula: R(t) = 1-S(t).

PS, propensity score; HR, hazard ratio; ITA, internal thoracic artery; NNT, number needed to treat

## Discussion

The main findings of our study are that:

1. Estimation of treatment effect modification is highly dependent on the survival model used. A negative interaction on a multiplicative scale (i.e. an interaction that corresponds to a treatment effect closer to one when age increases for example) can conversely translate into a similar treatment effect on an additive scale when the baseline hazard increases with the interaction covariate, as in the case with age.
2. On the other hand, absolute treatment effect on survival can be evaluated with an additive or multiplicative survival model. Importantly, the Cox model can efficiently quantify absolute risk reduction either in the whole sample or across subgroups. In addition, PS-adjusted data can easily be analyzed with either multiplicative or additive survival models while PS-adjusted absolute treatment effect on risk can be derived from either method. To the best of our knowledge, we provide the first report combining PS methods and additive hazard modeling, which could pave the way for a more frequent and homogenous reporting of absolute effects in observational studies.

### Assessment of treatment on an additive and on a multiplicative scale

In both observational and randomized studies, the association with survival is expressed in most cases, if not all, on a multiplicative scale as a hazard ratio. However, absolute risk reduction or increase, i.e. the difference in probability of an event at a given time, has been largely advocated as a more clinically useful measurement of effect [20–22]. In order to provide more clinically useful estimates of treatment effect in RCT, it is now recommended by the CONSORT statement to provide absolute effect measurements of treatment [23]. The STROBE statements regarding cohort studies recommend, when relevant, translating estimates of relative risk into absolute risk [24]. Additive interaction, often referred to as synergy, can be evaluated in most modeling settings, including relative risk models and logistic models [25]. However, clinical researchers often consider the absolute effect as a second line analysis, the significance of the association being predominantly investigated with a classical relative effect measurement. In contrast, as others [21], we consider that the absolute scale is the most important and informative measurement of treatment effect. The primary intention of clinicians and policy makers is to prevent as many (absolute) deaths as possible, and consequently target the highest absolute risk reduction.

In the present study, we demonstrate that the assessment of treatment effect modification is intrinsically highly dependent on the scale on which it is assessed. Herein, the treatment of interest was associated with a greater effect on survival in older patients on a relative scale (HR for interaction per additional year = 1.03, 95% CI: 1.00 to 1.06, p = 0.05) whereas it was equally associated with absolute hazard reduction across age subsets (delta for interaction per additional year = 0.10, 95% CI: -0.27 to 0.46, p = 0.61) due to a much higher baseline hazard of events in older patients. In essence, survival following an additive model will be identified as sub-multiplicative when assessed on a multiplicative scale. The mortality hazard specific to the pathology of interest (i.e. coronary disease in this instance) may be more important in older patients. In turn, the intervention of interest (i.e. bilateral ITA use) targeting the disease of interest would have an equal absolute effect in older patients despite its lower relative effect.

The interpretations of the results provided by the two models by a non-statistician or non-epidemiologist are very likely to differ. The non-methodologist analyst is likely to interpret the results of the Cox model as a greater beneficiary effect of treatment in the younger patients whereas the results of the additive model would be interpreted as a similar beneficiary effect in the older patients–or even a greater effect in patients >70 years old. These very different conclusions could translate into highly divergent decisions for clinicians who treat older patients: Attending clinicians would probably be less likely to allocate treatment to older patients with the results expressed as relative hazard than as difference of risk. Considering that the assessment of absolute treatment effect is intuitively impossible, we strongly advocate for its systematic straightforward quantification within the results of clinical publications. This point of view is in line with the work of other authors [10,26].

These large differences in interaction measurement in the additive and multiplicative scales have previously been described in the setting of epidemiological exposure[10]. However, translating this concept to therapeutic clinical research could be just as appropriate as in an epidemiological setting. Indeed, in the context of public health, it is primarily the policy makers who ultimately translate the results of a study into genuine changes in public health campaigns. Importantly, clinical trials were recently demonstrated to almost systematically use relative effect measurements for subgroup analyses [27].

In the present example, we further demonstrate that PS-adjusted absolute risk reduction can be obtained using a multiplicative survival model. Indeed, NNTs derived from PS-adjusted predicted survival using the Cox model were remarkably similar at the end of the follow-up period in patients aged < = 60 and in patients >70 (NNT = 27.7 and 20.0 in patients aged < = 60 at 8 and 10 years respectively vs. 27.7 and 22.0 in patients aged >70, Table 4), which is consistent with the similar hazard difference according to age yielded by the Aalen model.

Other tools could have been used to assess absolute treatment effect (attributable fraction [28] or risk [29]) or additive interaction (relative excess risk due to interaction [30,31]). Importantly, very simple tools can sometimes be useful to assess additive interaction, such as the direct comparison of probabilities, which we previously used in a re-analysis of published data of a clinical trial [1]. Yet, these other tools, from our point of view, provide less direct assessment of effects than the additive hazard models, and might appear less straightforward to readers. For instance, it is puzzling to assess risk differences (i.e. at a given time point) as absolute effect estimation whereas hazard ratios (i.e. for the entire follow-up) are used for relative effect estimation. In addition, the choice of the time point used to calculate risk difference is troublesome. In our opinion, the most convenient and homogenous approach is to estimate absolute treatment effect on the scale of rates (i.e. hazard), as for relative treatment effect estimation. Moreover, as we previously highlighted [1,2], risk differences are scarcely used. Using jointly additive and multiplicative hazard modeling could represent a convenient option to increase reporting on an additive scale. It would also increase the homogeneity of reporting of treatment effect on an additive and multiplicative scale. Similarly, additive modeling has been promoted to study dichotomous outcome variables [32]. However, the present work is one of the first to explore this strategy in the field of time to event analysis.

### Additive hazard models and propensity score analyses

Our study shows that PS-adjusted analyses can be easily performed using the additive model. However, stratification on matched groups is not possible with currently available software and/or packages performing additive models. We consequently had to perform a non-stratified analysis adjusted for PS. It is nonetheless hopeful that future developments of the timereg package of the R software will include stratification.

### Limitations

We could not formally assess the performance of the additive models fitted herein since these tools are not readily available in current statistical packages. However, we were able to assess the fit of the models using residuals as reported in S1–S3 Texts. In addition, survival curves derived from univariable additive models were very similar to those calculated from the Kaplan-Meier method, further suggesting acceptable adequacy of the additive hazard model to the data.

Treatment effect reporting was assessed in the setting of an observational study, which has some intrinsic caveats despite the use of propensity score. Further reports should investigate the use of additive hazard modeling in the setting of RCTs, which might benefit less from assessment of adjusted absolute effect quantification than observational studies. However, in RCTs, assigning the same level of priority to additive treatment effect reporting using both additive and multiplicative hazard modeling may still be useful to promote its use.

### Perspectives

Why is relative hazard estimation usually the sole measurement of treatment effect provided in clinical studies? One common answer is that, in most cases, relative treatment effect estimates are assumed to be constant across different baseline risks [33–35]. This assumption has recently been put forward by the PROGRESS 3 statement [35]. However, in contrast with this assertion, the treatment effect measured on a relative scale in our study was of greater strength in a low-event rate subset. In the present example, since there is a significant effect modification according to age in the Cox model, the latter model is therefore less parsimonious than the Aalen model. From a statistical standpoint, the Aalen model, in this setting, might be preferred. The assessment of treatment effect exclusively in the relative scale prevents assessing the magnitude of the treatment effect in a clinically useful manner, which could modify the care professional's assessment of patient benefit according to different age subsets. In our opinion, absolute treatment effect should be expressed at every step when reporting results. This is the main novelty of the present report, whereby the use of additive hazard modeling rather than other strategies could promote absolute treatment effect reporting and increase its homogeneity. As mentioned in the CONSORT and STROBE statements, we would be strongly in favor of providing, with the same level of visibility, absolute risk difference in addition to relative hazard. As consistently shown over the past years, even clinical trials often fail to provide assessment of the treatment effect on an absolute scale [6,36,37]. The absolute treatment effect, if expressed, is often translated into NNT. Unfortunately, NNTs are far from being systematically calculated even in prestigious medical journals [6]. In addition, the absolute effect, assessed through NNT or risk differences should not be limited to the overall population of a study. The assessment of treatment-effect modification should also be assessed on both the additive and relative scales. Taken together, all of these aspects strongly suggest that a measurement of treatment effect on an additive scale would help analysts to better evaluate treatment effect and treatment effect modification. Importantly, such absolute effect measurements can and should be evaluated in multivariable models in the setting of observational studies. It is the hope that the present work will help promote the use of absolute effect measurements, especially additive hazard modeling, in the future.

## Conclusions

We report herein an example of assessment of relative and absolute treatment effect using various methods. Irrespective of the method used, we found that lower treatment effect in older patients on a relative scale can conversely translate into a similar treatment effect on an additive scale due to large baseline hazard differences. Importantly, absolute risk reduction, either crude or adjusted, can be calculated from either multiplicative survival models. We advocate for a broader use of the absolute scale to assess treatment effect and treatment effect modification, regardless of the method used, in the setting of therapeutic clinical research, whether in RCTs or in observational studies. In addition, it is our belief that using additive hazard modeling along with multiplicative hazard modeling, putting relative and absolute treatment effect assessment on an equal plane (i.e. on the hazard level), could promote the reporting of absolute treatment effects.

## Supporting Information

S1 Text Validity assessment of the assumptions of the survival model. (DOCX)

S2 Text Assessment of the model fit of the constant hazard difference additive model and the proportional hazard Cox model for continuous variables. (DOCX)

S3 Text Formal presentation of the Additive hazard model and of the Cox model. (DOCX)
