# Supplementary material for: Quantification of Treatment Effect Modification on Both an Additive and Multiplicative Scale
Source: PLoS One. 2016 Apr 5;11(4):e0153010. doi: 10.1371/journal.pone.0153010 (PMC4821587; doi:10.1371/journal.pone.0153010)

**Supporting information 1**

**Validity assessment of the assumptions of the survival model.**

The proportional hazard Cox model makes the assumption that the hazard ratio remains constant over time whereas the constant hazard difference additive model makes the assumption that the difference in hazards remains constant over time. Each of these assumptions was assessed within the models constructed herein.

**I/ Cox model.**

To assess the validity of a constant hazard ratio over time, several methods can be used. A convenient approach is to use the cox.zph function of the Survival package of the R Software. We report below the output of the function used in a Cox model with treatment and propensity score as independent variables in the whole sample of our dataset. This function provides a visual assessment of Schoenfeld residuals, a modeling of the overall departure from 0 of Schoenfeld residuals over time and an overall test of deviation from constant hazard ratios over time. The visual assessment of the Schoenfeld residual graph and the p-value provided by the test both suggest that the hazard ratios are constant over time.


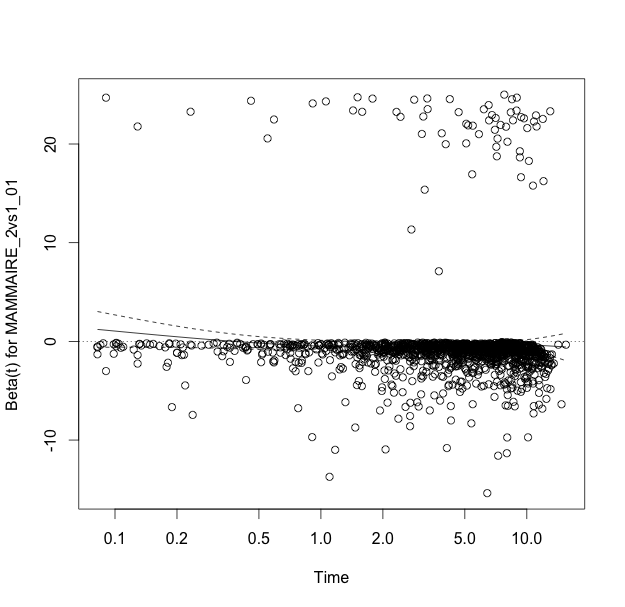


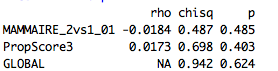


**II/ Additive model.**

To assess the validity of the assumption of a constant hazard difference over time, we need to perform the non-parametric form of the additive model. To achieve this, the Aalen function of the Timereg package should be used without the wrapper const() which indicates a Time-invariant regressor.

We report below the output of the function performed with treatment and propensity score as independent variables in the whole sample of our dataset. We can observe that the Kolmogorov-Smirnov test yields p values greater than 0.05.


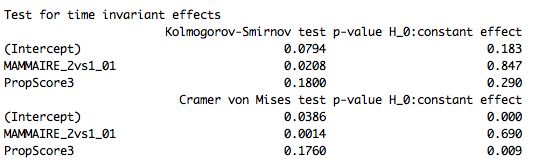


Upon further plotting the cumulative coefficients over time, we observe that, aside from the first 2 years during which cumulative coefficients remain very close to 0, there is a linear relationship between cumulative coefficients and time. This visual impression is concordant with the absence of rejection of the null hypothesis (constant effect over time) when using the Kolmogorov-Smirnov test.


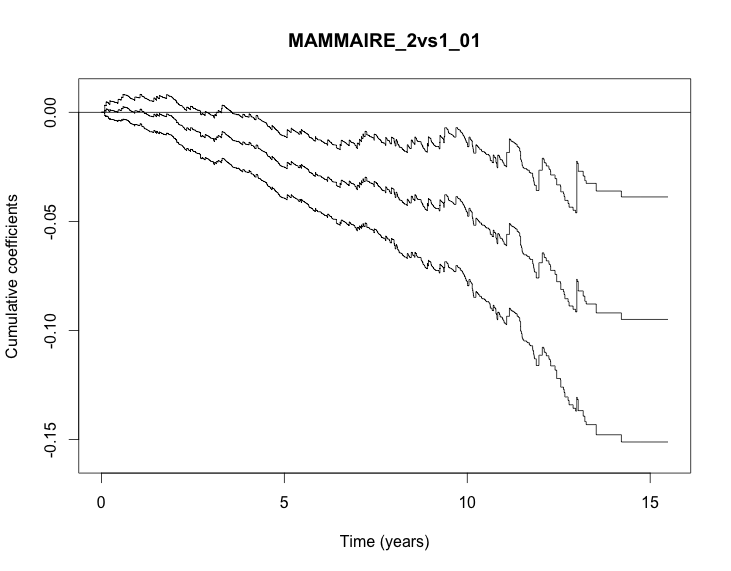

Supplement: S1 Text — (DOCX) [file pone.0153010.s001.docx]
