# Supplementary material for: Quantification of Treatment Effect Modification on Both an Additive and Multiplicative Scale
Source: PLoS One. 2016 Apr 5;11(4):e0153010. doi: 10.1371/journal.pone.0153010 (PMC4821587; doi:10.1371/journal.pone.0153010)

**Supporting information 2**

**Assessment of the model fit of the constant hazard difference additive model and the proportional hazard Cox model for continuous variables.**

The evaluation of cumulative martingale residuals is efficiently performed for both the additive and multiplicative models to model the functional form of continuous variables effect with the “Timereg” package of the R software.

When Propensity score was entered in the model as a continuous linear variable, important cumulative martingale residuals were identified both in the additive model (left panel) and the multiplicative model (right panel).


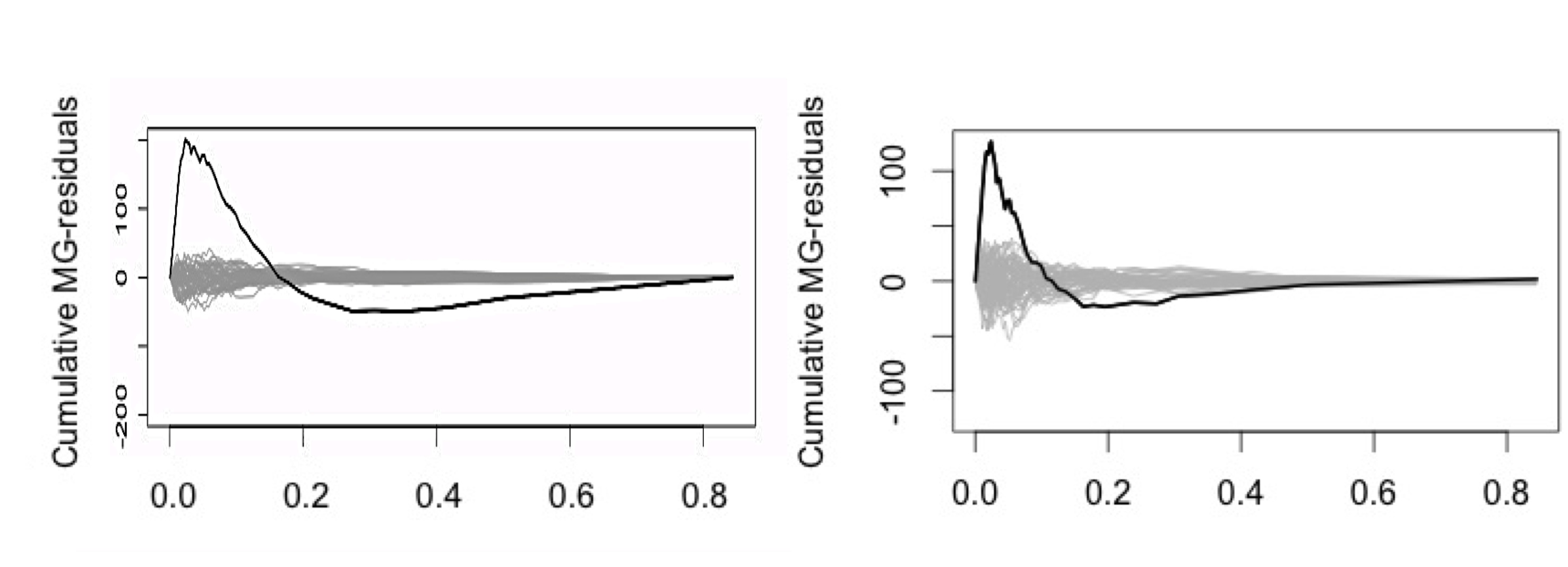


The cubic spline of the Propensity score was then used in the models. We constructed a model which included propensity score, age as a linear variable, the treatment of interest and the interaction between the treatment and age. Cumulative martingales residuals were no longer significantly different from zero in the additive model (left panel) and the multiplicative model (right panel).


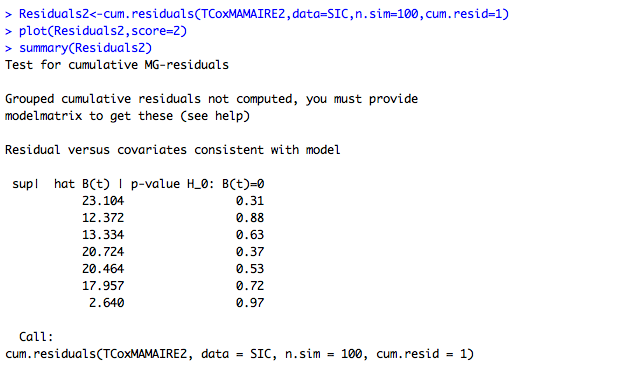

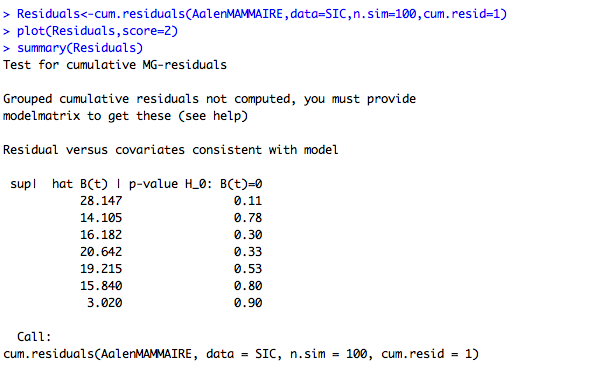


The visual inspection of cumulative martingales residuals confirmed an appropriate model fit. The cumulative residuals of the multiplicative model are provided below. The cumulative residuals of the additive model were similar.


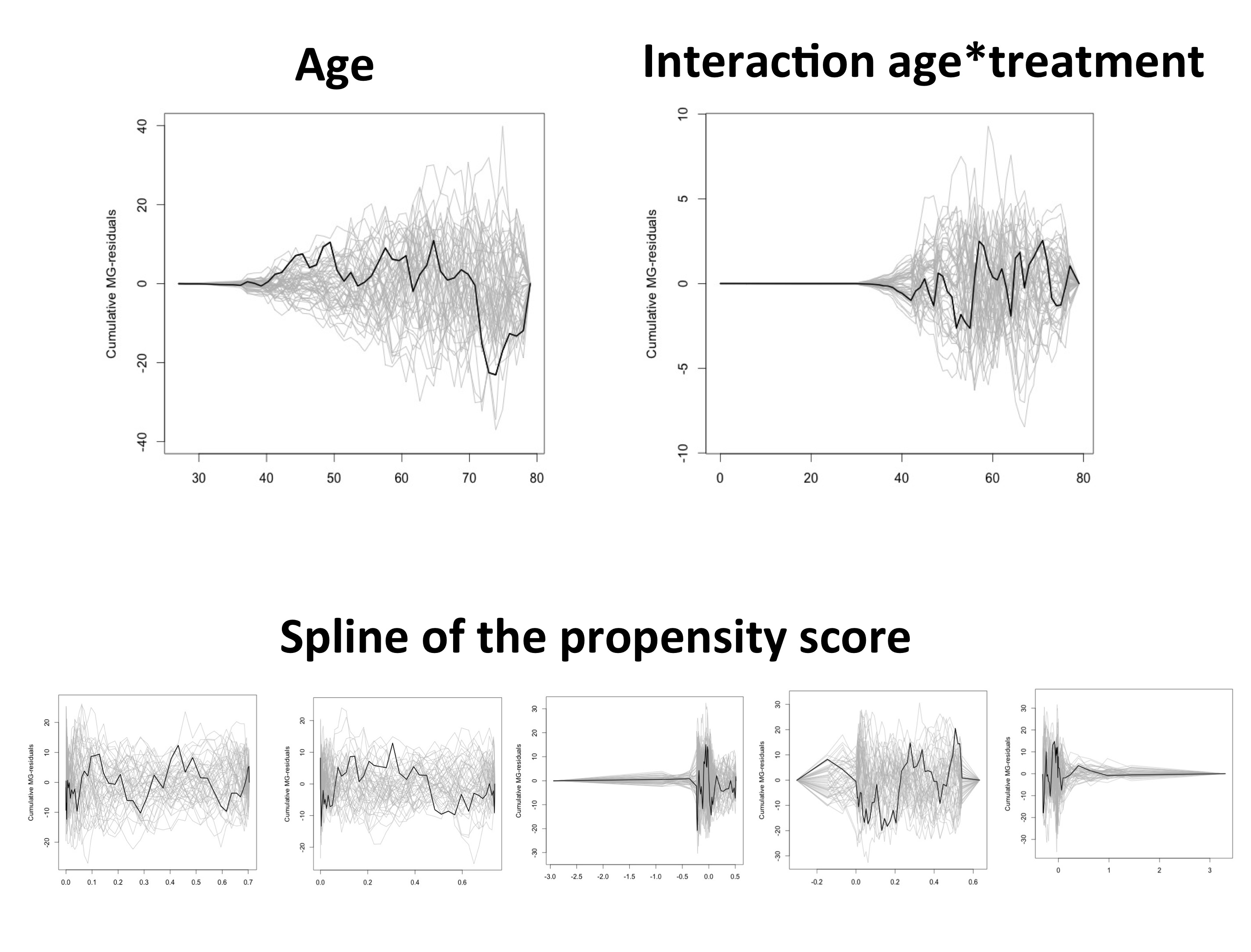


No significant interaction with time was identified in the additive model (left panel) and the multiplicative model (right panel).


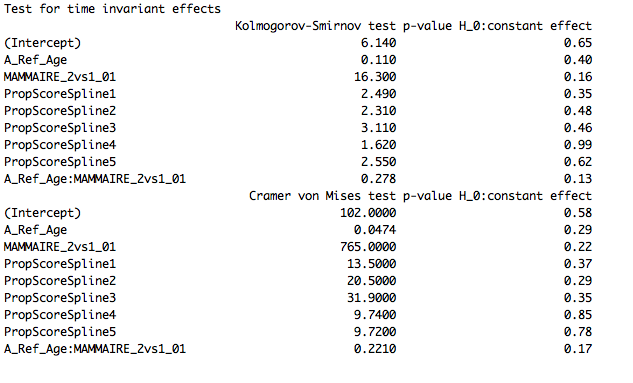

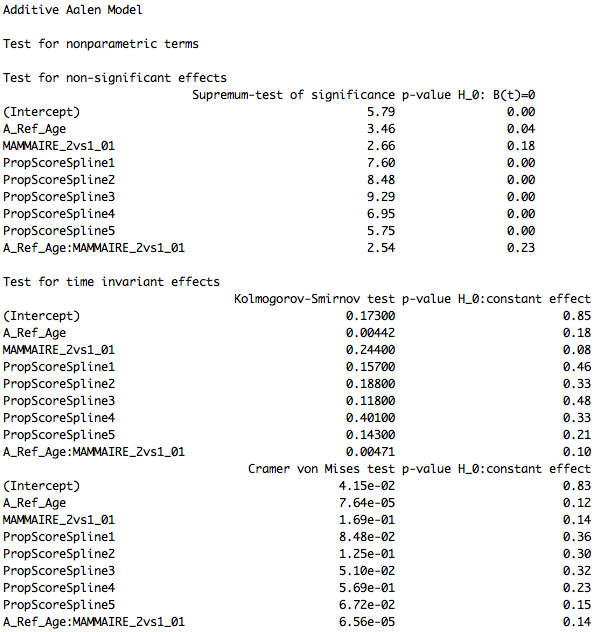

Supplement: S2 Text — (DOCX) [file pone.0153010.s002.docx]
