# Supplementary material for: Quantification of Treatment Effect Modification on Both an Additive and Multiplicative Scale
Source: PLoS One. 2016 Apr 5;11(4):e0153010. doi: 10.1371/journal.pone.0153010 (PMC4821587; doi:10.1371/journal.pone.0153010)
Supplement: S3 Text — (DOCX) [file pone.0153010.s003.docx]

**Supporting information 3**

**Formal presentation of the Additive hazard model and of the Cox model.**

**Additive hazard model:**

The constant hazard difference additive model can be written as follows:

$$\lambda\left(t\left|\text{X}\right.\right)=\lambda_{0}\left(t\right)+\sum_{k=1}^{p} \beta_{k}x_{k}$$

where

$\lambda\left(t,\text{ X}\right)$ is the hazard function of overall mortality conditionally on the vector of covariates X,

$\lambda_{0}\left(t\right)$ is the baseline hazard function,

$x_{k}$is the kth covariate of the vector of covariates included in the model.

$\beta_{k}$ is the regression coefficient, which quantifies the effect of the kth covariate on the baseline hazard as hazard difference.

In the additive model, interaction is assessed on an additive scale, and is expressed as additional (or subtracting) cases per N patient-year in the presence of another covariate. The “Timereg” package available for the R software is a very efficient tool for performing both the fully non-parametric Aalen model and the constant hazard difference model. Within this package, tests for time-independent effects and model fit assessment based on martingale residuals analysis can be easily performed.

In the constant hazard difference additive model, the survival probability, which is the complement of risk, can be written conditionally to the treatment variable A and to the adjustment covariate B as:

$$S\left( t|A,B \right)=exp\left( -\boldsymbol{\Lambda}\left( t|A,B \right) \right)=\exp\left[ -\left( \int_{0}^{t} \lambda_{0}\left( u \right) du+\left( \beta_{A}\times A+\beta_{B}\times B \right)\times t \right) \right]=S_{0}(t)\times\exp\left[ -\left( \beta_{A}\times A+\beta_{B}\times B \right)\times t \right]$$

where

t is the time considered to assess the survival probability,

Λ(t|A,B) is the cumulative hazard at time t, conditionally to treatment A and to covariate B,

$\beta$A and $\beta$B are the regression coefficients of treatment A and adjustment covariate B respectively, which represent the differences in hazard.

These survival probabilities can be extracted from the model while fixing the value of t, A and B using the Timereg package available from the R software.

**The Cox model**

The Cox proportional hazard model can be written as follows:

$$\lambda\left(t,X\right)=\lambda_{0}\left(t\right)\exp\left(\sum_{k=1}^{p} \beta_{k}x_{k}\right)$$

where

$\lambda\left(t,X\right)$ is the hazard function of overall mortality conditionally on the vector of covariates X,

$\lambda_{0}\left(t\right)$ is the baseline mortality hazard,

$x_{k}$is the kth covariate of the vector of covariates included in the model.

$\beta_{k}$is the regression coefficient, which quantifies the effect of the kth covariate on the baseline hazard. It is the logarithm of the hazard ratio, which quantifies the multiplicative effect of the covariate on the baseline hazard.

In the Cox model, the survival probability can be expressed using the same notation as that of the additive model, with the exception of $\beta$A and $\beta$B which are the log of the hazard ratios:

$$S\left( t|A,B \right)=\exp\left( -\int_{0}^{t} \lambda_{0}\left( u \right) du\times\exp\left( \beta_{A}\times A+\beta_{B}\times B \right) \right)={S_{0}(t)}^{\exp\left( \beta_{A}\times A+\beta_{B}\times B \right)}$$

These survival probabilities can be extracted from the model while fixing the value of t, A and B using the basehaz coxph object of the Survival package available from the R software.
